# Distinct and competing interneuron populations can generate fast and slow gamma in oscillatory models of CA1

**DOI:** 10.1186/1471-2202-16-S1-P119

**Published:** 2015-12-18

**Authors:** Stephen L Keeley, Andre A Fenton, John Rinzel

**Affiliations:** 1 Center for Neural Science, New York University, New York, NY 10003, USA; 2 Courant Institute of Mathematical Sciences, New York University, New York, NY 10012, USA

## 

Gamma oscillations are widely observed in the mammalian brain and are important markers for cognition and attention [1,2]. In CA1 of the hippocampus of freely moving rats, power in one of two distinct oscillatory bands in the gamma regime (fast gamma and slow gamma) is predominantly present at a given moment of time [3]. Here, we demonstrate that models of networks with competing interneuron populations with different post-synaptic effects can create distinct oscillatory regimes that mimic the observed oscillations of CA1. Our network formulation reflects the following facts: 1) The duration of post-synaptic effect of an interneuron strongly influences the frequency in biophysical models of gamma oscillations [4]. 2) The primary CA1 inputs from CA3 and the entorhinal cortex (EC) preferentially innervate interneurons of different subtype with different post-synaptic durations [5,6].

We show that a firing rate model with competing interneuron populations with different post-synaptic time-constants is sufficient to generate slow and fast gamma oscillations. We conclude that mutual inhibition between the modeled interneuron populations permits switching in a bistable regime between distinct fast and slow gamma states. We also find similar behavior in spike-based network models. Our models explicitly predict the following about CA1: 1) Different interneurons innervated by different upstream regions phase-lock to different gamma states. 2) One population of interneurons is silenced, and another is active during fast and slow gamma events. 3) Mutual inhibition between interneuron populations is necessary for spontaneous switching of gamma state. Using experimental electrophysiological data from awake behaving rodents, we find interneurons that satisfy conditions 1 and 2, and we show putative 'fast' and 'slow' gamma interneurons categorized by their tendency to fire and phase-lock with oscillatory events as measured by a nearby local field potential.

**Figure 1:**
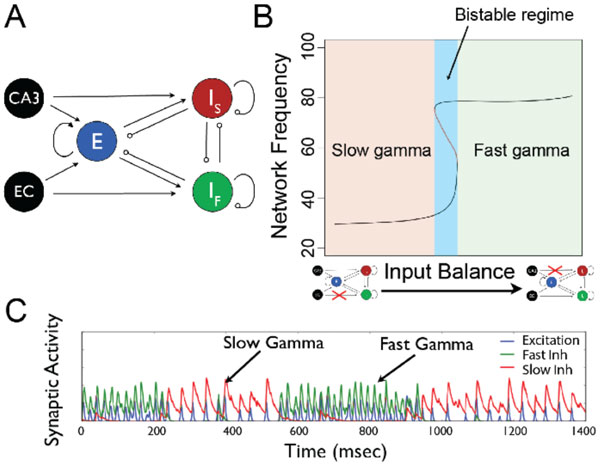
**A. Connectivity scheme for firing-rate two-gamma model**. CA3 and EC denote inputs, E denotes excitatory population, and I_F _and I_S _denote the interneuron populations with brief and long post-synaptic effects, respectively. **B**. Graph shows the oscillation frequency of the noise-free network across a range of input balance to I_S _and I_F_. Blue region indicates bistable regime with co-existing fast and slow oscillatory states. Such a bistable regime only exists with high I_S _- I_F _connectivity. **C**. Time courses of each population's synaptic variable show spontaneous switching between fast and slow gamma states due to additive noise in inputs to I_S _and I_F_.

Our 3-population firing rate model is schematized in Figure 1A. The dynamic variables are synaptic currents of an excitatory, fast inhibitory (I_F_) and slow inhibitory (I_s_) population; the firing rates are instantaneous functions of total input current. Fast excitation that interacts with inhibitory subpopulations supports oscillations. This interaction engages either one or both inhibitory subpopulations depending on I_S _- I_F _connectivity and input balance (Example in Figure 1B). This network oscillates at biophysically realistic frequencies given biophysically realistic network parameters. The fast inhibitory population, I_F _and slow inhibitory population, I_S _have post-synaptic time-constants of 5ms and 15ms, respectively. These roughly capture the diversity of post-synaptic inhibitory current time-courses of interneurons of different subtypes measured in CA1 [6]. Our firing rate model demonstrates that with sufficient mutual inhibition between inhibitory populations, the oscillating network bifurcates into two stable regimes that oscillate at roughly the same frequencies as the observed fast and slow gamma oscillations [3,7].

Previous experimental work suggests these two gamma oscillations reflect different information processing modes in the learning and memory system [7]. Our models provide a mechanistic understanding of these modes and posit a new oscillatory role for distinct interneurons in CA1. Moreover, our models describe general oscillatory behavior in networks with distinct interneuron populations.
